# The Implementation of a GPS-Based Location-Tracking Smartphone App in South Africa to Improve Engagement in HIV Care: Randomized Controlled Trial

**DOI:** 10.2196/44945

**Published:** 2023-05-19

**Authors:** Kate Clouse, Sandisiwe Noholoza, Sindiswa Madwayi, Megan Mrubata, Carol S Camlin, Landon Myer, Tamsin K Phillips

**Affiliations:** 1 Vanderbilt University School of Nursing Nashville, TN United States; 2 Vanderbilt Institute for Global Health Nashville, TN United States; 3 Division of Epidemiology and Biostatistics School of Public Health University of Cape Town Cape Town South Africa; 4 Department of Obstetrics, Gynecology & Reproductive Sciences University of California, San Francisco San Francisco, CA United States

**Keywords:** mobile health, mHealth, smartphone, mobile phone, HIV/AIDS, South Africa, pregnancy

## Abstract

**Background:**

Mobile health interventions are common in public health settings in Africa, and our preliminary work showed that smartphones are increasing in South Africa. We developed a novel smartphone app—CareConekta—that used GPS location data to characterize personal mobility to improve engagement in HIV care among pregnant and postpartum women living with HIV in South Africa. The app also used the user’s location to map nearby clinics.

**Objective:**

We aimed to describe the feasibility, acceptability, and initial efficacy of using the app in a real-world setting.

**Methods:**

We conducted a prospective randomized controlled trial at a public sector clinic near Cape Town, South Africa. We enrolled 200 pregnant (third trimester) women living with HIV who owned a smartphone that met the required specifications. All participants installed the app, designed to collect 2 GPS heartbeats per day to geolocate the participant within a random 1-km fuzzy radius (for privacy). We randomized (1:1) participants to a control arm to receive the app with no additional support or an intervention arm to receive supportive phone calls, WhatsApp (Meta Platforms, Inc) messages, or both from the study team when traveling >50 km from the study area for >7 days. In addition to mobility data collected daily through the phone, participants completed questionnaires at enrollment and follow-up (approximately 6 months post partum).

**Results:**

A total of 7 participants were withdrawn at enrollment or shortly after because of app installation failure (6/200, 3%) or changing to an unsuitable phone (1/200, 0.50%). During the study period, no participant’s smartphone recorded at least 1 heartbeat per day, which was our primary feasibility measure. Of the 171 participants who completed follow-up, only half (91/171, 53.2%) reported using the same phone as that used at enrollment, with the CareConekta app still installed on the phone and GPS usually enabled. The top reasons reported for the lack of heartbeat data were not having mobile data, uninstalling the app, and no longer having a smartphone. Acceptability measures were positive, but participants at follow-up demonstrated a lack of understanding of the app’s purpose and function. The clinic finder was a popular feature. Owing to the lack of consistent GPS heartbeats throughout the study, we were unable to assess the efficacy of the intervention.

**Conclusions:**

Several key challenges impeded our study feasibility. Although the app was designed to reverse bill participants for any data use, the lack of mobile data was a substantial barrier to our study success. Participants reported purchasing WhatsApp data, which could not support the app. Problems with the web-based dashboard meant that we could not consistently monitor mobility. Our study provides important lessons about implementing an ambitious GPS-based study under real-world conditions in a limited-resource setting.

**Trial Registration:**

ClinicalTrials.gov NCT03836625; https://clinicaltrials.gov/ct2/show/NCT03836625

**International Registered Report Identifier (IRRID):**

RR2-10.1186/s13063-020-4190-x

## Introduction

### Background

There are an estimated 7.5 million people living with HIV in South Africa, more than the number of people living with HIV in any other country [1]. The country adopted a universal test-and-treat antiretroviral therapy (ART) policy in 2016, allowing for the initiation of lifelong ART regardless of clinical criteria [2]. Despite the widespread availability of free ART and the known efficacy of the treatment for prevention [3-5], South Africa still had >200,000 new HIV infections and 51,000 deaths from HIV in 2021 [1].

Continuous engagement in HIV care is a known challenge in South Africa [6,7]. Pregnant women living with HIV are at an especially high risk of dropping out of HIV care, particularly during the postpartum period [8-12]. Our earlier work has explored the potential of mobility, particularly long-distance travel to the mother’s rural home, as a factor contributing to postpartum disengagement in care [13-15]. This work was limited in that it either required a retrospective analysis of existing data or relied on the self-reported mobility recall of participants still engaged in care.

Mobile health (mHealth) apps are frequently deployed in public health settings in Africa [16-19], and our preliminary research showed that smartphones are increasingly common among our target population of postpartum women living with HIV in South Africa [20]. Previous studies demonstrated the utility of aggregated cellular phone data in showing population mobility [21-24]. Furthermore, US-based studies demonstrated the feasibility of opt-in tracking of people at high risk of HIV [25,26]. Within this context, we developed a smartphone app that could track a participant’s location—with their permission—throughout late pregnancy and the postpartum period to characterize mobility prospectively and offer a support intervention to those who traveled.

### The CareConekta App

The CareConekta app was built through collaboration between the study team and Jembi Health Systems in Cape Town [27]. It followed an initial beta version developed and briefly tested in collaboration between the study team (KC, LM, and TKP) and Dr Martin Were of Vanderbilt University Medical Center’s Department of Biomedical Informatics and was based on qualitative preliminary research that explored attitudes toward mHealth interventions and possible concerns regarding location tracking among potential users [28,29]. The app uses the phone’s GPS signal to prospectively characterize mobility in real time, which is a real advancement in research that previously relied on retrospective analysis. Information on a participant’s location would allow the study—and later, clinic—staff to intervene without delay to link traveling individuals to health facilities in the new area, with assistance through phone calls or WhatsApp (Meta Platforms, Inc) messages. In addition, loaded with a national list of HIV care facilities that could be located on a map, the app acted as a clinic finder. We previously published the study protocol [27] and descriptions of the cohort [30] and screening process [31]. In this paper, we describe our primary outcome of the feasibility of implementing this app and the operational lessons learned by conducting an mHealth study in a resource-limited setting in South Africa.

## Methods

### Study Design and Dates

We conducted a prospective, unblinded randomized controlled trial at the Gugulethu Midwife Obstetric Unit (MOU), a public sector clinic providing integrated HIV and peripartum care for pregnant women near Cape Town, South Africa. Full details of the study design can be found in our published protocol [27], and details of the participant characteristics and follow-up can be found in the cohort profile [30]. In addition to characterizing mobility during pregnancy and the postpartum period, we designed the app to serve as a tool for engagement in HIV care for mobile women living with HIV. Thus, we randomized (1:1) participants to a control arm to receive the app with no additional support or an intervention arm to receive the app and supportive phone calls, WhatsApp messages, or both from the study team when meeting our threshold for traveling: >50 km from the study area for >7 days. The enrollment goal was 200 participants, which we anticipated would be an attainable goal given our study period and our objective of describing the feasibility, acceptability, and initial efficacy of the intervention. Enrollment began in December 2019 and ended in February 2021. There was a 6-month pause in recruitment from March to September 2020 due to the COVID-19 pandemic. The participant follow-up ended in November 2021.

### App Design Specifications

To characterize participant mobility during the study period, the CareConekta app was designed to collect 2 GPS location heartbeats per day. In addition, the app was built with a geographic list of health facilities in South Africa so that users could see a map of nearby facilities as they traveled. App connectivity and participant location (marked using anonymized study ID numbers) were viewable to the study team through a password-protected, web-based dashboard. To protect participant privacy, the location was made fuzzy by randomizing the location within a 1-km radius. The MOU was set as the home location from which to begin measuring movement. The mobility history was saved on an encrypted, password-protected server at a South African data center.

The app was available for free download through the Google Play Store (Google LLC) but required authentication and registration, so access was restricted to those enrolled in the study. CareConekta was designed such that it would cost the participant nothing: data costs associated with location tracking would be immediately reimbursed through reverse billing. Therefore, the cellular service of 1 of the 4 major mobile providers in South Africa was a requirement for eligibility. In the event of disconnection, the app was designed such that location data would be stored on the phone and uploaded as soon as connectivity resumed. However, reverse billing did not apply for app installation or version updates, so participants installed the app at the clinic using free Wi-Fi, and small data bundles were provided by the study staff for reinstallation and updates, when needed.

### Recruitment and Eligibility

Pregnant women were recruited during routine antenatal care at the MOU. Women were eligible if they were in the third trimester of pregnancy (≥28 weeks); aged ≥18 years; able to speak and understand isiXhosa (the predominant local language) or English; diagnosed with HIV at any time before enrollment; able to demonstrate basic smartphone-level literacy; and willing to participate in all aspects of the study, including randomization and mobility tracking. Eligible participants also needed to own a smartphone that met the technical requirements described in the subsequent section.

### Smartphone Technical Requirements

For the purpose of this study, a smartphone was defined as a mobile phone device with a touchscreen interface and internet and GPS capabilities. CareConekta was designed for phones using the Android operating system, version 5.0 or later. In our preliminary work, nearly 90% of the smartphones of the women approached for study participation used the Android system [20]. Eligible participants were required to subscribe to service (prepaid or contract) from 1 of the main 4 cellular providers in South Africa: Vodacom (Vodacom Group Limited), Cell C (Cell C Limited), Telkom (Telkom SA SOC Limited), or MTN (MTN Group Limited). At recruitment, the eligible participant needed to demonstrate that the phone could use GPS by opening a map app, such as Google Maps (Google LLC), and finding the current location. Finally, the phone needed to be capable of holding battery charge; phones were ineligible if they needed to be charged more than twice per day on average (by self-report). Details of those found to be ineligible during the screening assessment can be found elsewhere [31].

### App Installation and Operation

The app was installed at the study site by connecting the smartphone to the study’s Wi-Fi source. Because the app was available on the Google Play Store, all participants first needed a Google email address to download the app. For the app to function properly after installation, the GPS needed to remain enabled (with location allowed), and the phone needed to have some data or airtime available for reverse billing to work. Participants were asked to contact the study team if they needed to reinstall the app because of changing devices or uninstalling the app.

### Study Measures

Participant-reported data were collected at enrollment and follow-up—approximately 6 months post partum—directly into REDCap (Research Electronic Data Capture; Vanderbilt University) using tablet computers. REDCap is a secure, encrypted, and web-based software platform designed to support data capture for research studies [32]. Feedback on app installation experience was noted by the staff in REDCap at the end of the enrollment visit. App versions and participant contact attempts were maintained in study logs. Mobility data were exported from the CareConekta dashboard as CSV files.

### Analysis

We report counts and proportions for categorical variables and medians and IQRs for continuous variables. Data analysis was performed using SAS (version 9.4; SAS Institute). Open-ended responses were reviewed to identify key topics or themes and illustrative quotes.

### Ethics Approval

This study was approved by the institutional review boards of Vanderbilt University (reference 181640) and the University of California, San Francisco (237757), and the Human Services Research Committee of the University of Cape Town (659/2018). All participants signed a written informed consent form before enrollment, which included specific permission for location tracking.

## Results

### Installation Experience

App installation at enrollment was a highly variable experience. The most common reasons for slow experiences at installation were the need to delete items on the phone to make room for another app, the need to create a Google or Gmail account to use the Google Play Store, the need to update the Google Play Store app version, or problems sending and receiving heartbeats after installing the app. The following is a quote from the study staff’s notes about a particularly troublesome installation experience:

Had to create a Google account for participant and update Play store for app installation. Downloading took a while still. Had to let participant go and she came back a day later for installation; phone still taking forever. The controls get frozen, cleaned phone for efficiency but it still freezes. Finally, app installed and registered participant. Heartbeat data transmitted after all necessary settings adjusted.

### Investigator Withdrawals

In total, 7 participants were withdrawn from the study soon after enrollment owing to technical issues. Most (6/200, 3%) were withdrawn at the end of the enrollment visit because of an app installation failure that could not be resolved. In addition, 1 (0.5%) other participant was withdrawn for changing their phone to an ineligible phone within 2 weeks of enrollment.

### Participant-Reported Study Feasibility Measures

**Figure 1:**
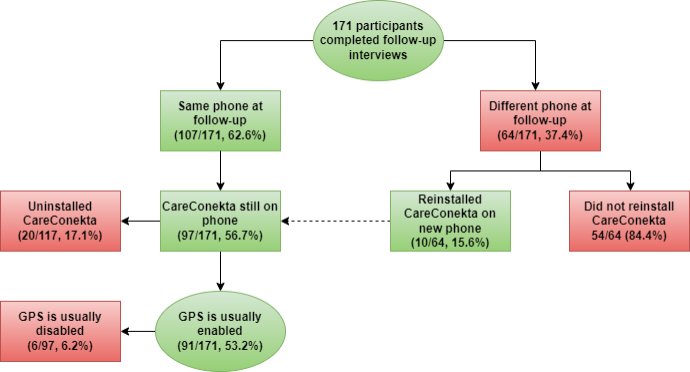
Number of participants who reported being able to use the CareConekta app at study follow-up. A total of 173 participants completed the follow-up interviews, but the first 2 participants completed the study before the variables presented here were added to the questionnaire. In total, 10 participants who had uninstalled the CareConekta app reinstalled it with the assistance of the study team and continue through the flowchart, as shown.

Figure 1 shows the feasibility measures of using the CareConekta app as designed, as reported by the participants at follow-up. Over one-third (64/171, 37.4%) of the participants reported that they were no longer using the smartphone in which the app was installed during enrollment. The top 3 reasons for changing phones were that the other phone stopped working (27/64, 42%), the other phone was lost (18/64, 28%), and the other phone was stolen (12/64, 19%).

Of the 64 participants who reported changing devices during the study, 10 (16%) reported that the CareConekta app was reinstalled on the new device. Among the 107 participants with the same phone at follow-up and the 10 participants who had reinstalled the app, 17.1% (20/117) reported that the CareConekta app had been uninstalled.

Of the 97 who still had the app on their phone, 6 (6%) participants reported that GPS was usually disabled on their phones. The reasons mentioned by those who reported disabling the GPS setting were that their phone did this automatically or that they turned it off to conserve the battery.

Deducting those who changed phones, those who uninstalled the app, and those who disabled GPS, only 53.2% (91/171) of the participants at the time of follow-up reported the possession of a phone that would operate the app correctly.

### Phone Sharing

Approximately one in seven (25/173, 14.4%) participants reported sharing their phone during the study. These participants most often shared their phone with one of their family members (12/25, 48%), their boyfriend or husband (11/25, 44%), or one of their friends who was not their boyfriend or husband (3/25, 12%). Of those who reported phone sharing, 80% (20/25) reported that they had their phone with them most of the day.

### Version Updates

From December 2019 to December 2020, the CareConekta app moved from version 1 to version 7. At one point, a version change (version 5 to 6) meant that the heartbeat data submitted by older versions were no longer received. From July to August 2020, participants were phoned, notified of the new app version, and assisted with updating the app over the phone (Google Play > CareConekta > update app). In cases where participants struggled to follow the steps over the phone, we offered an option for them to come to the study clinic—especially in cases where they were scheduled for an upcoming visit—where we would update the app for them in person. On a few occasions, to those who experienced difficulty over the phone, we sent the steps to follow via SMS text message or WhatsApp. The participants were offered data (100 MB) to update the app over the phone.

### Gaps in GPS Heartbeat Signals

A key feasibility measure was the successful transmission of at least 1 data location heartbeat per participant per day. Heartbeat data were observed daily by the study staff via the dashboard and periodically exported as a CSV file for analysis. During the study, we experienced periods when the dashboard was offline and data exports were unavailable, which resulted in missing data. Owing to difficulties with the dashboard and data exports resulting in substantial missing periods of data after March 19, 2021, our analysis of heartbeat data was conducted on all heartbeats received between the start of enrollment (December 2019) and March 2021. Table 1 shows the summary of gaps in heartbeats received during this period.

The daily GPS heartbeats of none of the participants were received without interruption. The range in heartbeat gaps was 2 to 273 days.

Among the 127 participants with gaps of ≥28 days, 55 (43.3%) were participants whose heartbeat transmission stopped altogether; the remaining 72 (57%) were women whose heartbeat transmission had long gaps but heartbeats resumed during the study period.

On the basis of 3302 heartbeats with GPS location, the median distance traveled from the study site was 2.4 (IQR 1.5-4.5) km. A total of 104 heartbeats (16 women) picked up a distance >50 km away. Only 3 (1.6%) out of 193 women were >50 km away for >7 days based on the heartbeat data, thus meeting our study definition of “travel.” Of the 3 women, 2 (67%) were in the control arm and received no additional action, and 1 (33%) was in the intervention arm; the participant in the intervention arm had a break in GPS when she reached a rural area in November 2020, but her GPS heartbeats resumed in January 2021, and the intervention protocol was followed.

In comparison, self-reported mobility during the follow-up visit indicated 37 trips lasting ≥7 days during the study period. Most of these trips were missed by the CareConekta app data.

**Table 1:** Gaps in GPS heartbeats received during the CareConekta study.

	Total (n=193), n (%)	Intervention (n=98), n (%)	Control (n=95), n (%)
One heartbeat received per day	0 (0)	0 (0)	0 (0)
Maximum gap in heartbeats: <7 days	11 (5.7)	7 (7.1)	4 (4.2)
Maximum gap in heartbeats: 7-13 days	15 (7.8)	8 (8.2)	7 (7.4)
Maximum gap in heartbeats: 14-20 days	15 (7.8)	7 (7.1)	8 (8.4)
Maximum gap in heartbeats: 21-27 days	11 (5.7)	5 (5.1)	6 (6.3)
Maximum gap in heartbeats: ≥28 days	127 (65.8)	62 (63.3)	65 (68.4)
No heartbeat received during analysis period	14 (7.3)	9 (9.2)	5 (5.3)

### Participant-Reported Reasons for GPS Gaps

From September 2020 to September 2021, our team attempted to contact all participants for whom a GPS heartbeat could not be detected. Contact attempts were made through phone call, followed by SMS text message, if needed. Over this 1-year period, participants could be contacted multiple times for a lack of heartbeat, and the result of the contact could differ each time. Therefore, we present the number of contact attempts and the primary reason reported for the GPS failure at the time of contact.

Out of the 454 contact attempts, 247 (54.4%) were unsuccessful, as the participants were unreachable or declined to talk. The reasons for GPS heartbeat loss gathered during the 207 (45.6%) successful contacts are listed in Table 2.

The most cited reason for why participants’ phones failed to send GPS heartbeat signals was a lack of data. Overall, 40.6% (84/207) of the contacts reported this reason. Over one-third of them (32/84, 38%) reported that they had purchased mobile data only for WhatsApp. The second most cited reason for signal failure was that the CareConekta app was uninstalled from their phone (28/207, 13.5%). Phone change often occurred because of sharing phones with family members and friends, and children were often blamed for the deletion of apps from the phone. In addition, 9.7% (20/207) of the participants reported no longer having a smartphone, whereas 6.3% (13/207) changed to a different smartphone that did not have the app installed. Other reasons for GPS failure included phone malfunctions or broken phones (14/207, 6.8%), malfunctions related to the CareConekta app (14/207, 6.8%), and the disabling of GPS (13/207, 6.3%).

**Table 2:** Primary reason for GPS heartbeat gap reported through 207 participant contacts.

Primary reason for GPS heartbeat gap	Value (n=207), n (%)
Lack of mobile data	84^a^ (40.6)
Uninstallation of the CareConekta app	28 (13.5)
Phone change: new phone is not a smartphone	20 (9.7)
Phone malfunction or broken phone	14 (6.8)
CareConekta app–related malfunction	14 (6.8)
Phone change: app not installed on new phone	13 (6.3)
Disabling of GPS	13 (6.3)
Unclear reason or failure of troubleshooting attempt	21 (10.1)

^a^32 (15.4%) participants mentioned having only WhatsApp data.

### Raffle

In November 2020, to encourage participants to keep the CareConekta app installed on their phone and the GPS function enabled throughout the study period, we implemented an incentive: a weekly raffle of one 200 MB data bundle (worth approximately US $4). Participants were eligible to enter the raffle if their phone sent GPS coordinates at least once a day in the prior week. Among all eligible participants, 1 winner per week was randomly selected. The weekly raffle incentive only applied to the participants (n=86) enrolled from November 2020 onward who had signed version 5.0 or later of the informed consent document. There was no limit to the number of times a participant could win the raffle.

From the 41-week period of November 18, 2020, to September 15, 2021, the weekly raffle was drawn 32 times. Four drawings were missed because the CareConekta dashboard was down, and we could not see the GPS data. Two drawings were missed because of holidays. There were no winners for 3 weeks because no participants were eligible. From the 32 drawings, there were 23 unique winners. The same participants won the raffle repeatedly because of the small number of participants who met the eligibility criteria of having consistent heartbeats. In total, 3 participants won the raffle 3 times each during this period. We found that the raffle incentive made no difference to the consistency of GPS heartbeats.

### Additional Technical Challenges

On multiple occasions, the staff-facing dashboard was either not accessible or not fully functional, which meant that the team was unable to view or download heartbeat data. During the early study period, these problems often resulted in app revisions and version updates. In some instances, when the dashboard was down, the app did not work either, and heartbeats were not transmitted or recorded. Although the app was originally specified to store heartbeats on the phone and transmit them to the server when the connection was restored, this did not happen. Similarly, the app was designed to be reverse billed so as to not cost participants data for using the app; however, some data or airtime was needed on the device for the app to initiate.

### Data Expenditure

Overall, for all their cell phone needs, participants reported spending a median of R51 (IQR 30-100; US $2.75, IQR US $1.60-5.40) for data per month, with a similar response for monthly spend on airtime: median R50 (IQR 29-100; US $2.71, IQR US$1.57-5.40). Cellular data in South Africa cost approximately R10 (US $0.50) per 50 MB.

### Participants’ Understanding of the CareConekta App

At follow-up, participants were asked, “If you were to explain to a friend what the CareConekta app does, how would you explain it?” Nearly all participants (167/170, 98.2%) mentioned the clinic finder feature:

I’d say it’s an app used to search for clinics when I travel to the Eastern Cape so I don’t suffer when I run out of medication. I’d simply just search for a clinic on the app.

Participant #103

It is an app that can help you find clinics near you, so you don’t say you did not go to the clinic because you did not know where it was.

Participant #58

Only 5 (2.9%) mentioned geolocation tracking or the app knowing the participant’s location, and 2 (1.2%) participants said that they did not know the app’s function. None of the participants mentioned the app notifications or staff contact via WhatsApp or phone.

### Participants’ Acceptability of the CareConekta App

Similar to the participants’ responses regarding their understanding of the app, most of the responses regarding what participants liked about using the app were related to the clinic finder feature:

I found it useful because I don’t have to look for clinics should I travel outside Cape Town. The app connects me to the clinics closest to me.

Participant #19

What I liked was that we had been looking for a pediatric clinic and got lost in a taxi, I then thought of this app, I used it and it showed me exactly where the clinic was.

Participant #029

Some responses indicated that the participants used the clinic finder for a general map too:

With this app I know for a fact I’d never get lost when I go somewhere. The app has a GPS function.

Participant #88

### Initial Efficacy of the Intervention

Although the initial efficacy of the intervention was a secondary aim of the study, we were unable to assess this because the app did not function as designed. Without receiving regular GPS heartbeats, the study team did not know when a participant was traveling; therefore, the intervention could not be initiated. During the study period, only 1 participant in the intervention arm was flagged as traveling, as defined in our protocol, and received the additional notifications, so it was not possible to assess a statistically meaningful difference between the study arms.

## Discussion

### Principal Findings

This is one of the first GPS-based mHealth studies—if not the first GPS-based mHealth study—targeted at improving HIV care in South Africa, and we found that several key challenges impeded its implementation. This study was designed to test the feasibility and acceptability of the CareConekta app and the initial efficacy of using it as an intervention to improve engagement in care among mobile women living with HIV. Although we were able to accomplish our primary aim of assessing the feasibility and acceptability of the intervention, we were unable to assess the efficacy of the intervention because we did not receive consistent location-tracking data. It is important to note that the app missed picking up on travel that was reported at follow-up. Although this is disappointing, we feel that the lessons learned from the implementation of this ambitious mHealth study are important and will be useful to other researchers considering mHealth interventions for low-resource settings. In designing this study, we made a conscious choice to assess our app under real-world conditions. We briefly considered providing phones—particularly to avoid bias against those who did not own phones and to guarantee a consistent technical level of device—but decided that this would not allow us to interpret the real-world applicability of our results. Similar decisions were made against providing data to all participants. Thus, our results can be viewed as representing implementation in real-world conditions.

We developed an initial beta version of the CareConekta app and implemented it in a proof-of-concept trial in 2017. We enrolled 11 participants at the same study site. Among the 11 participants, app installation failed for 7 (64%) individuals. Because the app team was US-based, some requirements of the app did not align with the capabilities of many of the phones in use in South Africa, and we were also unable to offer real-time technological support in the event of installation difficulty. The importance of a local app development team, with available technology support, was one of the key lessons learned from this early work. We were also committed to collaborating with a local development company that works with and knows the mHealth agenda of the South African Department of Health; we wanted to be well poised for broader implementation if our app was successful. In the proof-of-concept trial, we were able to install the app on 4 participants’ phones and deploy the app for 3 months. Of the 4 participants, 1 (25%) lost her phone after approximately 1 month, but the other 3 (75%) produced heartbeats at least weekly, often daily, during the 3-month period. This sufficiently proved the concept for us to proceed with this study.

In this study, during the 15 months of intensive data monitoring, no participant had GPS heartbeats every day without interruption, which is a key indicator of study feasibility. Despite specifically designing the app such that mobility data would be stored on the app in the event of interruptions in data, the app did not function correctly and did not provide the missing data. Our attempts to troubleshoot lost GPS signals unexpectedly required substantial staff time; indeed, at least 1 staff member phoned participants every week for a year to ask about missing heartbeats. Most participants did not respond, or if they did, they requested a later callback and then still did not respond. CareConekta was designed to reverse bill, which meant that even if the mobile device had no airtime and no data, the app would still be fully functional. However, through implementation, we found that if a participant had no data at all, the app would not work. That is, for reverse billing to work properly, a small amount of data was first required. This became a major stumbling block for implementation, as the top reason cited by participants for missing GPS heartbeats was a lack of data. In addition, we received numerous reports of purchasing only WhatsApp data, a product that was unfamiliar to the researchers at the time of study design but appears to have grown in popularity during the course of our study. Although the lack of electricity was not mentioned as a reason for lost GPS heartbeats, the study period coincided with regular periods of load shedding—scheduled electricity blackouts to conserve power in South Africa—which would have impacted participants’ ability to keep their phones charged. Future mHealth studies will be wise to consider the high likelihood of the lack of data and electricity during study implementation.

Even as early as installation, difficulties arose in using the app. From our preliminary research, we knew that over 90% of our participant population used Android-based phones [20]. However, downloading and installing the app from the Google Play Store required a Google-authenticated email address, which not every participant had before enrollment. The availability of space on the phone for Google Play Store updates and the CareConekta app was also a challenge. Even after extensive troubleshooting and creating space, some phones that seemed to meet our technical specifications were still unable to successfully install CareConekta, and we had to withdraw 6 participants because of app installation failure. Despite being designed to collect heartbeats only twice per day, the app collected location data multiple times a day. This created a challenge for analysis and may have contributed to battery drain on devices.

Overall, we found that the participant acceptability of CareConekta was high, but we view this finding with caution because it does not align with the numbers of participants who reported losing phones and uninstalling the app or the high frequency of lost GPS heartbeats. It is possible that social desirability bias to report a positive experience to the interviewer influenced responses. In the follow-up responses, all participants understood the participant-facing function of the app—the clinic finder—but seemed to forget or not understand the passive geolocation tracking, despite the great efforts made to be explicit about location tracking at the time of informed consent. Future mHealth intervention developers should note that patient-facing features may be the ones that will be most understood and remembered among participants.

The proportion of phone sharing reported at follow-up (15%) is consistent with that reported at the time of enrollment (14%). This frequency of sharing is similar to another recent mHealth study in South Africa that found 11% phone sharing [33]. The possibility of phone sharing and potential lack of confidentiality should be considered when designing future mHealth studies in this setting. Relatedly, participants often reported that children who shared their phones were the ones responsible for uninstalling the app.

Ours is not the first smartphone-based study in South Africa to experience substantial feasibility challenges. A South African study conducted from 2015 to 2017 [34] was unable to meet its sample size requirements because 90.2% (n=3187) of the 3540 potential participants were ineligible at screening because of they did not have an Android phone (n=2100, 59.3%), their phone was not working (n=506, 14.3%), or they had a wrong Android version or inadequate RAM or mobile data (n=581, 16.4%). Screening for our study is described elsewhere [31] and did not encounter similar challenges, but the implementation of our study did encounter similar issues with the lack of data, and inadequate RAM may have caused the installation challenges we experienced, as apps and photos sometimes needed to be deleted to make space for the CareConekta app. A different South African study from 2019 found that 13% of the eligible participants were unable to download the study app on their Android-based phone, 6% were unable to scan a barcode, and 3% were unable to complete app registration [33]. During implementation, the authors noted reports of a lack of data access and lost or broken phones. MomConnect, a nationwide pregnancy registry service in South Africa, demonstrated broad success by sending simple SMS text messages but noted that network timeouts and failures were frequent, resulting in 1 in 4 users dropping out of the registration process prematurely, and recommended that platforms such as WhatsApp be adopted to encourage flexibility in messaging [35].

To our knowledge, this is the first mHealth intervention to use location-based participant tracking in a real-world setting in South Africa. Interventions such as this are becoming increasingly common in the United States and Europe. For example, in a study focused on travel health published in 2016, a total of 101 Swiss adult travel clients planning to travel to Thailand for <5 weeks were provided a smartphone equipped with an app to passively monitor their location and administer a daily questionnaire [36]. The authors found that the app was feasible and acceptable, but 10% (n=10) of the participants had technical difficulties, and 16% (n=16) dropped out during the brief follow-up period. In a US-based study of patients who were chronically ill published in 2018, a total of 27 participants were provided with a smartphone and location-tracking watch; 6 (22%) participants dropped out before the end of the 28-day study period, some owing to the inability to use the devices [37]. Of note, the investigators offered participants a financial incentive (up to US $100) to upload their data to the study. A US-based study published in 2021 provided smartphones to 30 individuals experiencing homelessness, who were followed for 4 months, with the goal of alerting their community-based team that the participants had entered hospital or emergency care [38]. The authors found that 6 (20%) participants were withdrawn after reporting their second study-provided smartphone stolen, and overall, only 19% of the GPS data aligned with hospital data, primarily owing to participants not having the smartphone with them during the visit, the smartphone being switched off, and gaps in GPS technology. Compared with our study, these studies had smaller sample sizes, shorter follow-up periods, very different settings, and provided smartphones or financial incentives; however, it is interesting to note that similar challenges were encountered in implementation. Despite the setbacks experienced in our study and in others, given the rise in off-the-shelf location-tracking apps in recent years and the clear importance of mobility in health, we anticipate that more mHealth interventions will focus on participant movement. Indeed, the massive *All of Us* campaign in the United States plans to incorporate location-based data from smartphones and wearable devices, including location, cardiac rate and rhythm, and respiratory rate [39].

Some of the problems experienced with app implementation may have been avoided through clearer communication between the research team and app development or technical team. Although we thought that we had very close communication, some aspects were lost in translation. It is critically important that the research team investigating any mobile app includes someone who fully understands the technical specifications and requirements and can liaise with or translate the research vision to the app development team, as well as caution the research team on potentially problematic areas of design.

Our study has some clear limitations, primarily that the app did not function as designed because of a combination of user actions and app malfunction. Another limitation is that we conducted the study at a single site, thus potentially limiting the generalizability of our results. However, given that we attempted to mimic real-world conditions, we anticipate that our study population will be similar to other adults attending public health clinics in South Africa.

However, we feel that many important lessons learned through this experience will be useful to other researchers and make the effort worthwhile. One strength of our study is that it was among the first to develop, implement, and clearly document experiences with a GPS-based location-tracking mHealth app in a low-resource setting, particularly in a real-world setting. Thus, our findings are meaningful, even if our intervention was not successful. In addition, we report on several technical factors traditionally unreported in the literature but critical to the feasibility of mHealth interventions.

### Conclusions

In conclusion, we did not demonstrate the feasibility of using a GPS-based tracking app to characterize mobility and improve engagement in HIV care. Our most common problems that contributed to failure were a lack of mobile phone data, app uninstallations, phone changes, and missing heartbeat data. We are far less motivated to create a novel app in our future research endeavors and instead will use tools that people already are using, particularly WhatsApp, which all of our participants reported as their favorite app [31]. Although some of our participants had no data for other apps, they were buying data specifically for WhatsApp. By using apps that are already essential to participants’ lives, researchers developing future studies can become better poised to ensure continuous engagement with the apps and decrease the likelihood of the apps being uninstalled. Careful translation of research aims into app design is essential in the development of future studies. In addition, the mHealth landscape is changing rapidly, and it is possible that Wi-Fi and internet availability will increase in the future, thus overcoming some of the challenges we experienced. We anticipate that mHealth interventions will continue to proliferate in resource-limited settings, such as our study setting, and our study results may offer guidance and words of caution for unanticipated challenges.

## Appendix

Multimedia Appendix 1 CONSORT-eHEALTH checklist (V 1.6.1).

## Abbreviations

ART: antiretroviral therapy
mHealth: mobile health
MOU: Midwife Obstetric Unit
REDCap: Research Electronic Data Capture
